# Association between Use of Spironolactone and Risk of Stroke in Hypertensive Patients: A Cohort Study

**DOI:** 10.3390/ph16010057

**Published:** 2022-12-30

**Authors:** Xintian Cai, Nanfang Li

**Affiliations:** Hypertension Center, Xinjiang Hypertension Institute, National Health Committee Key Laboratory of Hypertension Clinical Research, Key Laboratory of Xinjiang Uygur Autonomous Region, Xinjiang Clinical Medical Research Center for Hypertension Diseases, People’s Hospital of Xinjiang Uygur Autonomous Region, Urumqi 830001, China

**Keywords:** spironolactone, stroke, hypertension, cohort study, primary prevention

## Abstract

Objective: to investigate the relationship between the use of spironolactone and the risk of stroke in hypertensive patients. Methods: a total of 2464 spironolactone users and 12,928 non-users were identified (unmatched original cohort), and 1:1 matched pairs of 2461 spironolactone users and 2461 non-users based on propensity scores were created (propensity-score-matched cohort). Results: In the unmatched original cohort, the unadjusted analysis showed that the use of spironolactone was associated with a lower risk of total stroke (HR, 0.71; 95% CI, 0.61–0.84; *p* < 0.001), which was sustained in the adjusted analysis. According to stroke type, the association was with ischemic strokes (propensity-score-adjusted HR, 0.71; 95% CI, 0.59–0.85; *p* < 0.001) and hemorrhagic ones (propensity-score-adjusted HR, 0.63; 95% CI, 0.45–0.88; *p* = 0.008). Similar results were shown in the propensity-score-matched cohort. The results of the subgroup and sensitivity analyses were consistent with those of the primary analysis. The dose–response analysis demonstrated a dose-dependent association of spironolactone with a lower risk of stroke in hypertensive patients. Conclusions: The use of spironolactone was associated with a significantly lower risk of stroke events in hypertensive patients. Further research, including prospective randomized clinical trials, is needed to validate our findings.

## 1. Introduction

Stroke has become a worldwide public health problem [1]. In China, stroke is one of the leading causes of death and long-term physical and cognitive impairment [2]. According to the Global Burden of Disease Study 2019, there were 3.94 million new cases of stroke in China. The incidence rate of stroke increased by 86.0% from 1990, and the mortality rate increased by 32.3% from 1990 [1,3]. There are multiple major risk factors for stroke [4]. Some are not modifiable, such as age, gender, and race, while others, including lifestyle factors, may be modifiable [4,5,6]. The major modifiable risk factor for stroke is hypertension [7]. Therefore, a better understanding of additional modifiable risk factors for stroke has important clinical implications, especially in patients with hypertension. However, traditional risk factors (hypertension, diabetes, smoking, and dyslipidemia) do not explain all the risks of stroke. Several investigations have confirmed that they should also be partially attributed to the inappropriate release of aldosterone and/or the abnormal activation of mineralocorticoid receptors (MRs) [8,9,10].

Numerous studies have reported that the incidence of stroke is significantly higher in patients with aldosteronism than in patients with essential hypertension [11]. Similarly, it has been noted that the risk of stroke in patients with elevated plasma aldosterone appears to be independent of blood pressure [12]. Hyperaldosteronism and/or activation of MRs leads to endothelial damage, resulting in premature coronary artery disease, left ventricular hypertrophy, and proteinuria, all of which increase the propensity for stroke [11,13,14]. In patients with normal or high sodium intake, hyperaldosteronism and/or activation of MRs leads to oxidative stress and endothelial dysfunction in the cerebral circulation and has been identified as a predictor of stroke or transient ischemic attack [13,14]. In addition, animal models suggest that hyperaldosteronism and/or activation of MRs has deleterious effects on cerebrovascular comorbidity, independent of blood pressure and hypokalemia [15,16]. MR antagonists (MRAs), highly effective agents in the treatment of refractory hypertension, also reverse the adverse effects (sodium retention, potassium loss, endothelial dysfunction, vascular inflammation, myocardial hypertrophy, and fibrosis) of hyperaldosteronism and/or activation of MRs, leading to reduced oxidative stress and collagen deposition and improved perfusion of cardiac and brain tissue [17,18,19]. 

Spironolactone is a non-specific MRA and the only MRA currently approved for clinical use in mainland China. Spironolactone is effective for lowering blood pressure (BP) when used as a monotherapy or combination therapy [17]. In several clinical studies, spironolactone has been shown to prevent target organ damage and effectively reduce the risk of cardiovascular events and all-cause mortality in high-risk populations [20,21,22]. However, the use of spironolactone as an antihypertensive agent remains low. Current guidelines consider spironolactone as a second-line treatment for essential hypertension (except for hypertensive patients with secondary aldosteronism, where it is a first-line treatment), which substantially limits its use. Further large-scale studies demonstrating its effectiveness and end-organ protection are necessary before spironolactone can be elevated as a first-line treatment option for hypertension. To date, there are no large-scale studies demonstrating the role of spironolactone on stroke events in hypertensive patients. To address this, we conducted a population-based, propensity-matched, retrospective cohort study to assess whether spironolactone is associated with a reduced risk of stroke in patients with hypertension.

## 2. Results

### 2.1. Study Population

We identified 15,392 participants who met the inclusion criteria, of whom 2464 (16.0%) received spironolactone treatment. Before the propensity score matching, there were differences between the two groups on several variables (Table 1). By using propensity score matching, 2461 participants using spironolactone were matched with 2461 non-users. After matching, the absolute standardized differences were less than 0.1 for all variables, indicating minimal differences between the two groups (Table 1 and Figure S1).

### 2.2. Spironolactone Use and Stroke Risk in the Unmatched Original Cohort

**Figure 1:**
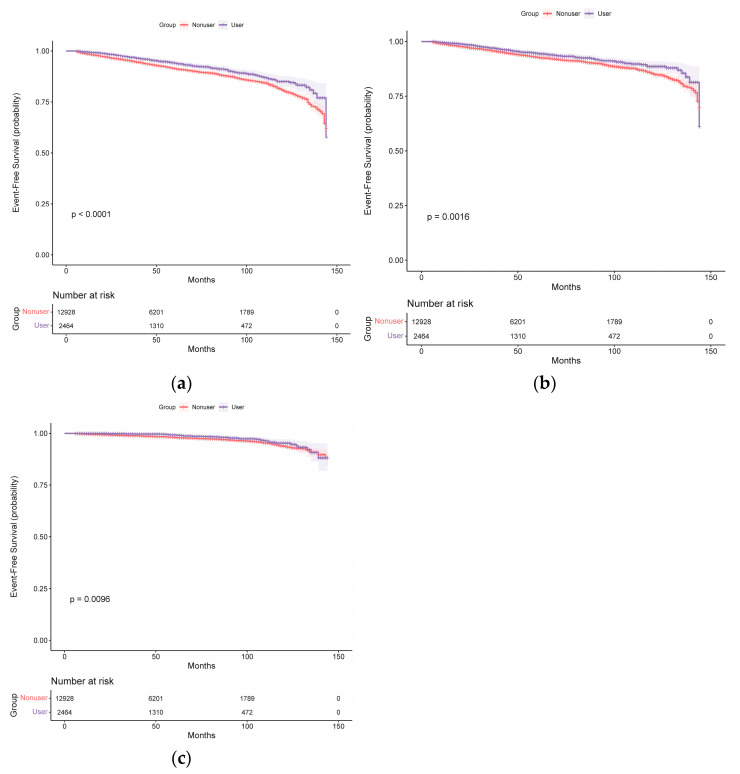
Kaplan–Meier curves for (**a**) total stroke, (**b**) ischemic stroke, and (**c**) hemorrhagic stroke in the spironolactone user and non-user groups (before propensity score matching).

During a median follow-up period of 48 months (IQR, 25–81 months), 1279 participants had at least 1 primary outcome event. The Kaplan–Meier survival curves for the primary outcome events are presented in Figure 1, and the cumulative event rates are shown in Table 2. The primary outcome event rates were 13.80 (11.80–16.05) events per 1000 person-years in patients taking spironolactone and 18.96 (17.86–20.11) events per 1000 person-years in those not taking spironolactone. The risks of experiencing total stroke events (unadjusted HR, 0.71; 95% CI, 0.61–0.84; *p* < 0.001) (Figure 1a), ischemic stroke events (unadjusted HR, 0.75; 95% CI, 0.63–0.90; *p* = 0.002) (Figure 1b), and hemorrhagic stroke events (unadjusted HR, 0.64; 95% CI, 0.46–0.90; *p* = 0.010) (Figure 1c) exhibited a significant decrease in patients taking spironolactone compared with those not taking spironolactone. 

After adjustment, the risk of experiencing total stroke events remained significantly decreased in patients taking spironolactone (multivariable-adjusted HR, 0.69; 95% CI, 0.59–0.81; *p* < 0.001 and propensity-score-adjusted HR, 0.68; 95% CI, 0.58–0.80; *p* < 0.001) (Table 2). After adjusting for confounders, the HR for total stroke events was not altered. The risk of ischemic stroke events was significantly lower in patients taking spironolactone than in those not taking spironolactone (multivariable-adjusted HR, 0.71; 95% CI, 0.60–0.85; *p* < 0.001 and propensity-score-adjusted HR, 0.71; 95% CI, 0.59–0.85; *p* < 0.001). The risk of hemorrhagic stroke events was also lower in patients taking spironolactone (multivariable-adjusted HR, 0.66; 95% CI, 0.47–0.92; *p* = 0.014 and propensity-score-adjusted HR, 0.63; 95% CI, 0.45–0.88; *p* = 0.008).

### 2.3. Spironolactone Use and Stroke Risk in Propensity-Score-Matched Cohort

**Figure 2:**
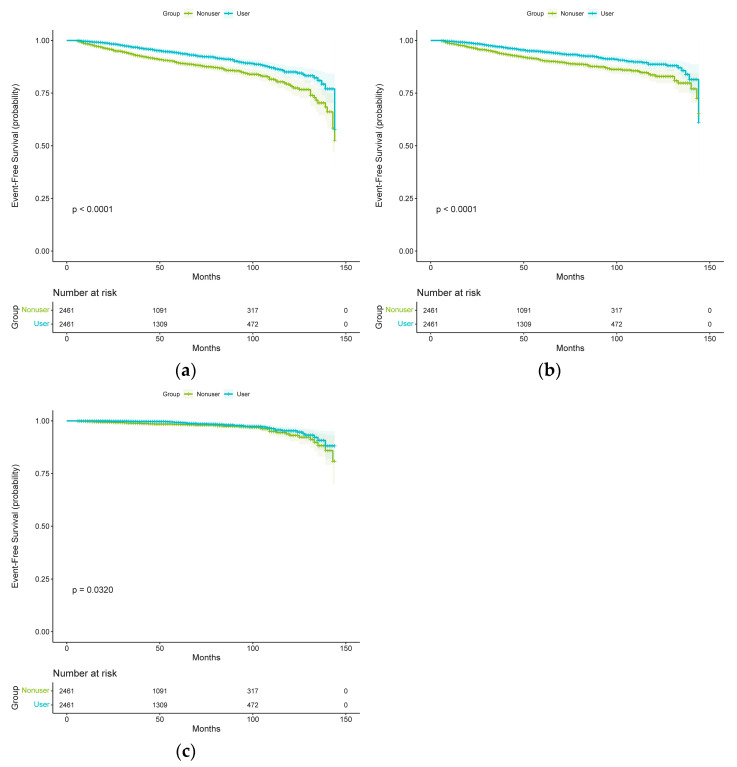
Kaplan–Meier curves for (**a**) total stroke, (**b**) ischemic stroke, and (**c**) hemorrhagic stroke in the spironolactone user and non-user groups (after propensity score matching).

In the propensity-score-matched cohort, 412 patients experienced at least 1 total stroke event during the follow-up period. Figure 2 demonstrates the corresponding curves in the propensity-score-matched cohort with results similar to those observed in the unmatched original cohort. Log-rank tests support the existence of significant differences among the different subgroups of spironolactone exposure. Among the propensity-score-matched patients, the risk of total stroke events was significantly lower in patients taking spironolactone than in those not taking spironolactone (multivariable-adjusted HR, 0.60; 95% CI, 0.49–0.73; *p* < 0.001 and propensity-score-adjusted HR, 0.59; 95% CI, 0.49–0.72; *p* < 0.001) (Table 2). Among the propensity-score-matched patients, the risks of ischemic stroke (multivariable-adjusted HR, 0.61; 95% CI, 0.49–0.76; *p* < 0.001 and propensity-score-adjusted HR, 0.60; 95% CI, 0.49–0.75; *p* < 0.001) and hemorrhagic stroke events (multivariable-adjusted HR, 0.63; 95% CI, 0.42–0.96; *p* = 0.033 and propensity-score-adjusted HR, 0.64; 95% CI, 0.42–0.97; *p* = 0.037) were also significantly lower in patients taking spironolactone than in those not taking spironolactone (Table 2).

### 2.4. Subgroups and Sensitivity Analyses

**Figure 3:**
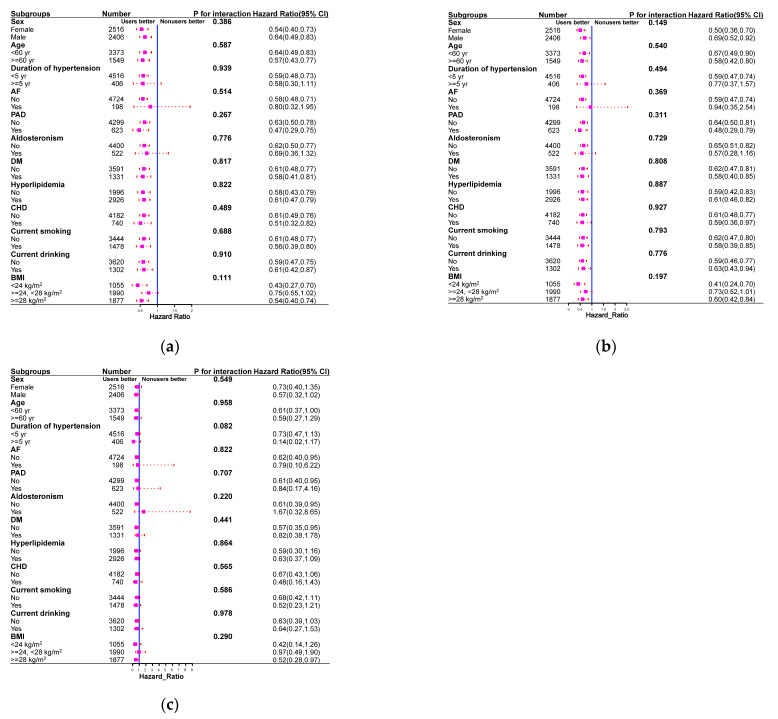
Subgroup analysis comparing spironolactone users with non-users in terms of the risks of total stroke (**a**), ischemic stroke (**b**), and hemorrhagic stroke (**c**) in propensity-score-matched cohort.

The reduction in HRs for total stroke events, ischemic stroke events, and hemorrhagic stroke events in spironolactone users was generally consistent in all subgroups (Figure 3). Furthermore, no significant interactions were observed between the use of spironolactone and the use of other drugs (all *p*-values > 0.05) (Figure S2). Similar patterns of association were observed after excluding individuals older than 80 years at baseline (Table S1), excluding total stroke events that occurred 1 year before follow-up (Table S2), and considering all-cause mortality as a competing risk (Table S3). The use of spironolactone was statistically significant in reducing total stroke events, ischemic stroke events, and hemorrhagic stroke events according to different propensity score matching ratios (Figure S3), possibly overcoming the type 2 error caused by 1:1 matching. Finally, the E values calculated to assess the robustness of the results against potentially unmeasured confounders are summarized in Table 2. The E values (point estimates) observed for the association between the use or non-use of spironolactone and total stroke events, ischemic stroke events, and hemorrhagic stroke events ranged from 1.21 to 2.12 (Table 2). The E values indicated that residual confounding due to potentially unmeasured confounding factors is probably moderate.

### 2.5. Dose and Cumulative Duration of Spironolactone Use and the Risk of Outcome Events

**Figure 4:**
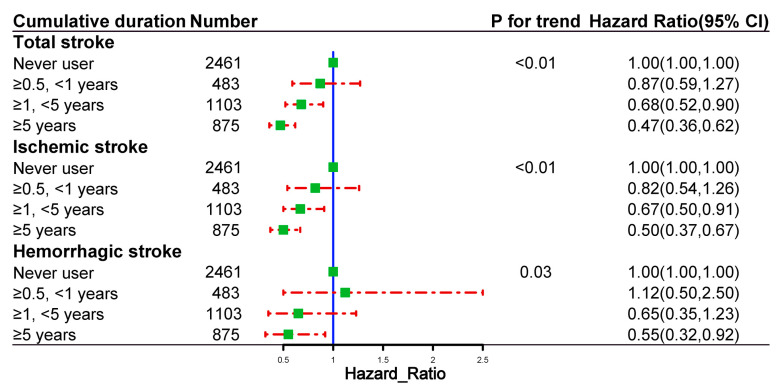
Dose–response relationships between the cumulative duration of spironolactone use and the risk of outcome events in propensity-score-matched cohort.

**Figure 5:**
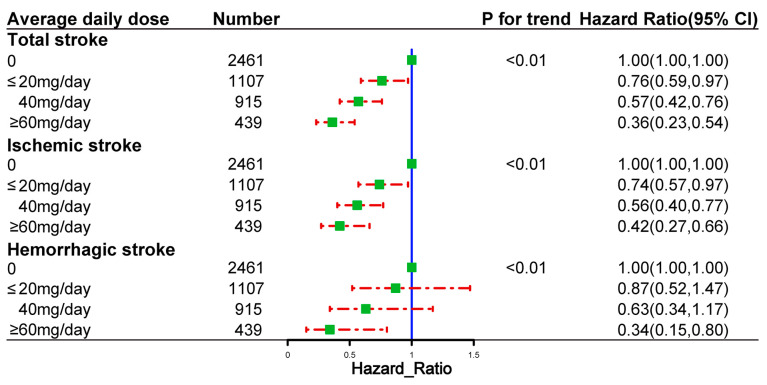
Dose–response relationships between the average daily dose of spironolactone and the risk of outcome events in propensity-score-matched cohort.

Figure 4 shows the risk of outcome events based on the cumulative duration of spironolactone use. The risks of total stroke, ischemic stroke, and hemorrhagic stroke decreased with the increasing cumulative duration of spironolactone use. Figure 5 demonstrates the risk of outcome events based on the average daily dose of spironolactone. The risks of total stroke, ischemic stroke, and hemorrhagic stroke decreased as the average daily dose of spironolactone was increased.

### 2.6. Safety Outcome

The incidence of hyperkalemia was slightly higher in the spironolactone use group than in the non-use group (multivariable-adjusted HR, 1.22; 95% CI, 0.79–1.88; *p* = 0.365 and propensity-score-adjusted HR, 1.23; 95% CI, 0.80–1.90; *p* = 0.342). Nevertheless, there was no significant relationship between the duration of spironolactone consumption and the risk of hyperkalemia (all *p*-values > 0.05). Users with an average daily dose of 60 mg/day, however, had a significantly higher risk of hyperkalemia than non-users (multivariable-adjusted HR, 1.83; 95% CI, 1.04–3.22; *p* = 0.035 and propensity-score-adjusted HR, 1.87; 95% CI, 1.07–3.28; *p* = 0.029) (Table S4).

## 3. Discussion

In this retrospective cohort study, we found that spironolactone use was associated with a reduced risk of stroke in hypertensive patients. This association was robust across multiple regression models, even after controlling for potential bias by propensity score matching, in subgroups stratified by age, sex, BMI, AF, PAD, aldosteronism, DM, CKD, HF, hyperlipidemia, CHD, duration of hypertension, smoking, and drinking habits. In addition, the results of this study suggested that spironolactone use in hypertensive patients was associated with a decreased risk of stroke in a dose–response pattern. Spironolactone has long been an extremely widely used drug in clinical practice for the treatment of refractory hypertension, chronic heart failure, and cirrhosis. In this study, we report the beneficial effects of spironolactone on the prognosis of stroke in hypertensive patients. To our knowledge, our clinical study is the first to assess the relationship between spironolactone use and the risk of stroke in hypertensive patients by analyzing large cohort data. These results may also have implications for the treatment of hypertension. 

Stroke has become a worldwide public health problem [23]. In China, stroke is the leading cause of death [2]. One of the major risk factors for stroke is hypertension [24]. Notably, since more than 75% of strokes are first occurrences, it is imperative to seek effective primary prevention strategies for stroke in China and worldwide [25]. Spironolactone is a well-established treatment modality for patients with myocardial infarction suffering from hypertension, heart failure, and left ventricular systolic dysfunction [20,21,22]. To date, few clinical studies have directly explored this issue. The lack of clinical data does not mean that spironolactone does not affect stroke. Clinically, there is a strong association between aldosterone and stroke; patients with primary aldosteronism and elevated plasma aldosterone levels are at a significantly increased risk of stroke compared with hypertensive patients without elevated aldosterone [26,27,28]. Preclinical evidence indicates that MR activation leads to a worse prognosis after stroke [29,30]. In a DOCA-induced hypertension model in rats, MR activation led to remodeling of the cerebrovascular system, resulting in increased vascular stiffness [31]. In addition, both Dorrance et al. and Rocha et al. showed that MR antagonists reduced stroke incidence/size and improved survival in an experimental model of stroke-prone spontaneously hypertensive rats maintained on a 1% physiological saline and stroke-prone diet [32,33]. Other studies have shown that spironolactone increases vascular tone in the middle cerebral artery (MCA) and improves vascular architecture by increasing vessels’ lumen and outer diameters in male but not female spontaneously hypertensive stroke-prone rats [34,35]. In contrast, another study showed that MR antagonism during the development of hypertension had the opposite effect on the MCA. It leads to a reduction in lumen diameter, which may be detrimental to vascular function and stroke outcomes, and an improvement in vasodilation [36]. In an experimental thrombosis model, aldosterone enhances thrombosis, whereas spironolactone reverses this effect [37,38]. In conclusion, these studies suggest that spironolactone may help reduce the risk of stroke in patients with hypertension.

The present study has several notable strengths. The large sample size increased the strength of the evidence concerning the biological effects of spironolactone. In addition, the results of interest were related to long-term clinical effects. In previous studies, available data of this nature were scarce. In actuality, among hypertensive patients, our study was the first to assess the potential association between spironolactone and the risk of stroke as a primary endpoint. Furthermore, the longer follow-up in this study than in usual clinical trials helped assess the long-term benefit. A longer follow-up was essential to determine whether this effect was temporary or persistent over time. Finally, the robustness of our findings was confirmed by different statistical methods, including propensity score matching, subgroup analysis, and sensitivity analysis. Thus, confounders and biases could be eliminated to the greatest extent possible, and the relevant findings were considered reliable. 

Despite these strengths, some limitations apply. First, because this was an observational study, we were unable to determine causality in the findings. Second, despite accounting for many important confounders, we cannot exclude residual confounding by unmeasured or unknown confounders. Therefore, large prospective randomized controlled trials are needed to elucidate the role of spironolactone in patients with hypertension. Third, physician preference for spironolactone use may lead to selection bias in the analysis. Fourth, prescription data do not show whether patients took the medication as prescribed. Therefore, the adherence to these regimens is unknown. Fifth, we determined the clinical comorbidities of each patient using ICD-10 codes, which may include over- or underdiagnosed conditions. Finally, given the small sample sizes of specific subgroups, the non-significant association between spironolactone use and the risk of stroke in some subgroups may simply be due to low statistical efficacy.

## 4. Materials and Methods

### 4.1. Study Design and Participants

We conducted an observational cohort study of hypertensive patients. De-identified patient information, such as ICD-10 diagnosis codes, prescription drug information, and lab test results, were collected from electronic medical records. Between 1 January 2010 and 31 December 2021, 18,609 hypertensive patients in total were polled. We restricted the group of chosen patients to adults by excluding individuals under the age of 18. We also excluded participants who were lost to follow-up or who had less than 6 months of follow-up. Furthermore, we excluded patients with any history of spironolactone use, stroke, and other disease states such as severe hepatic and/or renal insufficiency, systemic inflammatory disease, and malignancy at baseline. Finally, 15,392 patients were included (Figure S4). Our study complied with the Declaration of Helsinki and was approved by the hospital ethics committee (People’s Hospital of Xinjiang Uygur Autonomous Region). Due to the study’s retrospective nature, no additional informed consent was required. Moreover, this study adheres to the STROBE guidelines.

### 4.2. Variables

Patient demographics and comorbidities were extracted from electronic medical records. These included age, sex, duration of hypertension, body mass index (BMI), BP, heart rate, and comorbidities (diabetes mellitus (DM), hyperlipidemia, atrial fibrillation (AF), coronary artery disease (CHD), peripheral arterial disease (PAD), and aldosteronism). Laboratory and prescription data were collected using an automated data extraction system. The laboratory data included alanine aminotransferase (ALT), aspartate aminotransferase (AST), gamma-glutamyl transferase (GGT), total cholesterol (TC), triglyceride (TG), high-density lipoprotein cholesterol (HDL-C), low-density lipoprotein cholesterol (LDL-C), hemoglobin A1c (HbA1c), fasting plasma glucose (FPG), plasma aldosterone concentrations (PAC), and high-sensitivity C-reactive protein (hsCRP). The estimated glomerular filtration rate (eGFR) was calculated using the CKD-EPI equation [39]. The prescription data included antihypertensive drugs and other co-medications (statins, aspirin, and antihyperglycemic drugs). Details of the concomitant medications are shown in Table S5. Spironolactone users were defined as patients who took spironolactone for more than 180 days during the follow-up period. The start date was defined as the first prescription date, and the stop date was defined as the last prescription date plus the last prescription period. The definition criteria also apply to users of other drugs.

### 4.3. Follow-Up and Outcomes

The primary outcome was the first stroke (ischemic or hemorrhagic), which could be nonfatal or fatal. Secondary outcomes included the first ischemic stroke and first hemorrhagic stroke. Hyperkalemia was deemed a safe outcome. We defined hyperkalemia using an ICD-10 code E87.5, or a plasma/serum potassium (K+) level of ≥ 5.5 mmol/L from either inpatient and/or outpatient. Outcomes of events since participants enrolled in the study at baseline were determined through medical records, contact with local disease and death registries, or examination of the national health insurance system. Patients were followed from the date of enrollment to the end of the observation period, defined as the date of the last follow-up visit, the date of the first appearance of any study outcome, the date of death, or the end of the study period (31 December 2021).

### 4.4. Statistical Analysis

The quantitative variables are expressed as means (standard deviation) in the case of a normal distribution or medians (interquartile range) otherwise. Categorical variables are expressed as numbers (percentages). Patients were classified into two groups based on their use of spironolactone (users versus non-users). Given the differences in characteristics between the two groups of eligible participants (Table 1), propensity score matching (PSM) was used to identify a cohort of patients with similar characteristics. The precise details on the PSM are provided in the Supplementary Material. Absolute standardized differences (ASD) were estimated for all covariates before and after matching to assess pre-match imbalance and post-match balance. An ASD > 0.1 was interpreted as a meaningful difference for a given covariate. The number of stroke cases divided by the total follow-up time (person-years) was used to calculate the stroke incidence rate. Kaplan–Meier curves were plotted for the event-free probabilities of spironolactone users and non-users in the unmatched and matched cohorts. Log-rank tests were used to assess the differences between users and non-users. We checked the proportional hazards assumption and found no evidence that it was violated. In the primary analysis, hazard ratios and their 95% confidence intervals for different subgroups of spironolactone users and non-users were created with three Cox regression models: (1) unadjusted model; (2) multivariable Cox model (all covariates in Table 1 were considered independent variables); and (3) adjusted for propensity score. The analyses were performed in both the unmatched original cohort and the matched cohort. Further subgroup analyses were performed to assess the consistency of the observed treatment effects on outcomes at the level of different subgroup variables. The outcomes for the subgroup analysis were total stroke events, ischemic stroke events, and hemorrhagic stroke events. The subgroup variables were age (dichotomized by 60 years), sex, BMI (dichotomized by 24 and 28 kg/m^2^), AF, PAD, aldosteronism, DM, chronic kidney disease (CKD), heart failure (HF), hyperlipidemia, CHD, duration of hypertension (dichotomized by 5 years), smoking, and drinking habits. We also performed some sensitivity analyses in the matched cohort to assess the robustness of our study results. First, we excluded strokes that occurred within one year before the follow-up to assess potential reverse causality. Second, we excluded participants over the age of eighty. Then, in addition to the 1:1 propensity score matching, we performed three additional matching ratios (1:2, 1:3, and 1:4). In addition, a Fine-Gray competing risk regression was employed to treat non-stroke deaths as competing risk events. Finally, E values were applied to evaluate unmeasured confounding. Dose–response relationships were also investigated in this study. A multivariable Cox model was used to explore the association between the dose and cumulative duration of spironolactone use and the risk of outcome events. Analyses were conducted using R version 4.0.2. A *p*-value of less than 0.05 was considered significant.

## 5. Conclusions

In summary, this retrospective cohort study provides the first evidence that long-term spironolactone use reduces the risk of stroke in patients with hypertension. Our results endorse the need for prospective randomized clinical trials to study the effects of spironolactone in patients with hypertension.

## Figures and Tables

**Table 1:** Characteristics of the study participants.

	Unmatched Original Cohort			Propensity-Score-Matched Cohort		
Characteristic	Non-User Group	User Group	ASD	Non-User Group	User Group	ASD
No. of participants	12928	2464		2461	2461	
Age, year	51.55 ± 12.22	53.22 ± 11.64	0.14	53.20 ± 12.30	53.23 ± 11.63	0.00
Male, %	7005 (54.18%)	1202 (48.78%)	0.11	1206 (49.00%)	1200 (48.76%)	0.00
Current smoker, %	3824 (29.58%)	734 (29.79%)	0.00	746 (30.31%)	732 (29.74%)	0.01
Current drinker, %	3449 (26.68%)	665 (26.99%)	0.01	639 (25.97%)	663 (26.94%)	0.02
Duration of hypertension, %			0.04			0.04
<5 year	11918 (92.19%)	2246 (91.15%)		2273 (92.36%)	2243 (91.14%)	
≥5 year	1010 (7.81%)	218 (8.85%)		188 (7.64%)	218 (8.86%)	
Hemodynamics						
Heart rate, bpm	80.78 ± 10.37	80.85 ± 10.41	0.01	80.84 ± 10.56	80.85 ± 10.41	0.00
Diastolic blood pressure, mmHg	89.25 ± 14.58	89.04 ± 14.57	0.01	88.94 ± 14.36	89.04 ± 14.58	0.01
Systolic blood pressure, mmHg	144.54 ± 21.29	144.06 ± 20.74	0.02	143.80 ± 20.95	144.07 ± 20.75	0.01
Body mass index, kg/m^2^	27.10 ± 3.86	27.05 ± 3.91	0.01	27.07 ± 3.82	27.05 ± 3.91	0.01
Laboratory data						
Alanine aminotransferase, U/L	24.00 (15.00–35.36)	24.30 (15.27–35.89)	0.03	24.00 (15.00–34.34)	24.29 (15.28–35.90)	0.05
Aspartate aminotransferase, U/L	21.16 (16.19–28.00)	21.00 (16.40–28.13)	0.01	21.00 (16.00–27.96)	21.00 (16.41–28.15)	0.01
Gamma-glutamyl transferase, U/L	28.13 (17.71–42.00)	29.04 (18.47–43.12)	0.07	28.71 (17.79–42.00)	29.00 (18.47–43.14)	0.06
Estimated glomerular filtration rate, mL/(min*1.73 m^2^)	96.96 ± 18.49	96.24 ± 19.91	0.04	96.32 ± 19.37	95.49 ± 20.14	0.04
Total cholesterol, mmol/L	4.52 ± 1.01	4.49 ± 1.00	0.04	4.51 ± 1.01	4.49 ± 1.00	0.03
Triglyceride, mmol/L	1.60 (1.10–2.38)	1.63 (1.12–2.45)	0.05	1.92 ± 1.36	2.00 ± 1.57	0.06
High-density lipoprotein cholesterol, mmol/L	1.08 ± 0.29	1.07 ± 0.28	0.02	1.08 ± 0.29	1.07 ± 0.28	0.04
Low-density lipoprotein cholesterol, mmol/L	2.79 ± 0.84	2.72 ± 0.82	0.08	2.72 ± 0.86	2.72 ± 0.82	0.01
Hemoglobin A1c, %	6.12 ± 1.24	6.14 ± 1.29	0.01	6.17 ± 1.24	6.14 ± 1.29	0.03
Fasting plasma glucose, mmol/L	5.37 ± 1.82	5.41 ± 1.87	0.02	5.34 ± 1.91	5.41 ± 1.87	0.04
Plasma aldosterone concentration, ng/dL	16.51 ± 7.05	16.38 ± 7.04	0.02	16.48 ± 6.87	16.38 ± 7.04	0.01
High-sensitivity C-reactive protein, mg/L	2.68 (0.99–6.55)	2.64 (1.01–6.97)	0.02	2.86 (1.56–6.36)	2.90 (1.20-7.34)	0.02
Comorbidity						
Chronic kidney disease, %	412 (3.19%)	143 (5.80%)	0.13	122 (4.96%)	143 (5.81%)	0.04
Heart failure, %	209 (1.62%)	118 (4.79%)	0.18	98 (3.98%)	118 (4.79%)	0.04
Atrial fibrillation, %	285 (2.20%)	87 (3.53%)	0.08	112 (4.55%)	86 (3.49%)	0.05
Peripheral artery disease, %	1318 (10.19%)	292 (11.85%)	0.05	332 (13.49%)	291 (11.82%)	0.05
Aldosteronism, %	476 (3.68%)	264 (10.71%)	0.27	261 (10.61%)	261 (10.61%)	0.00
Diabetes mellitus, %	3165 (24.48%)	671 (27.23%)	0.06	661 (26.86%)	670 (27.22%)	0.01
Hyperlipidemia, %	7489 (57.93%)	1472 (59.74%)	0.04	1456 (59.16%)	1470 (59.73%)	0.01
Coronary heart disease, %	1736 (13.43%)	365 (14.81%)	0.04	376 (15.28%)	364 (14.79%)	0.01
Co-medications						
Statins, %	5612 (43.41%)	1158 (47.00%)	0.07	1134 (46.08%)	1156 (46.97%)	0.02
Aspirin, %	8307 (64.26%)	1653 (67.09%)	0.06	1641 (66.68%)	1652 (67.13%)	0.01
Calcium channel blockers, %	9997 (77.33%)	1972 (80.03%)	0.07	2018 (82.00%)	1970 (80.05%)	0.05
ACEI/ARB, %	9616 (74.38%)	1768 (71.75%)	0.06	1717 (69.77%)	1766 (71.76%)	0.04
Beta blockers, %	4880 (37.75%)	969 (39.33%)	0.03	957 (38.89%)	967 (39.29%)	0.01
Other diuretics, %	1411 (10.91%)	325 (13.19%)	0.07	307 (12.47%)	325 (13.21%)	0.02
Insulin, %	1116 (8.63%)	227 (9.21%)	0.02	220 (8.94%)	227 (9.22%)	0.01
Oral antidiabetic agents, %	2117 (16.38%)	439 (17.82%)	0.04	388 (15.77%)	438 (17.80%)	0.05

Data are presented as either a mean (± standard deviation), median (interquartile range), or proportion (%). ASD, absolute standardized difference; ACEI, angiotensin-converting enzyme inhibitor; ARB, angiotensin II receptor blocker.

**Table 2:** Associations between the use of spironolactone and the risk of outcome events.

	Unmatched Original Cohort			Propensity-Score-Matched Cohort		
	Non-User Group	User Group	E Value	Non-User Group	User Group	E Value
**Total stroke**						
No. of events (%)	1110 (8.6%)	169 (6.9%)		244 (9.9%)	168 (6.8%)	
IR per 1000 person-years (95% CI)	18.96 (17.86, 20.11)	13.80 (11.80, 16.05)		23.09 (20.28, 26.18)	13.73 (11.73, 15.97)	
Unadjusted HR (95% CI)	1.00 (reference)	0.71 (0.61, 0.84)	2.17	1.00 (reference)	0.59 (0.48, 0.72)	2.78
Multivariable-adjusted HR (95% CI)	1.00 (reference)	0.69 (0.59, 0.81)	2.26	1.00 (reference)	0.60 (0.49, 0.73)	2.72
Propensity-score-adjusted HR (95% CI)	1.00 (reference)	0.68 (0.58, 0.80)	2.30	1.00 (reference)	0.59 (0.49, 0.72)	2.78
**Types of stroke**						
**Ischemic stroke**						
No. of events (%)	877 (6.8%)	139 (5.6%)		200 (8.1%)	138 (5.6%)	
IR per 1000 person-years (95% CI)	14.98 (14.00, 16.00)	11.35 (9.54, 13.40)		18.92 (16.39, 21.73)	11.28 (9.48, 13.33)	
Unadjusted HR (95% CI)	1.00 (reference)	0.75 (0.63, 0.90)	2.00	1.00 (reference)	0.60 (0.48, 0.74)	2.72
Multivariable-adjusted HR (95% CI)	1.00 (reference)	0.71 (0.60, 0.85)	2.17	1.00 (reference)	0.61 (0.49, 0.76)	2.66
Propensity-score-adjusted HR (95% CI)	1.00 (reference)	0.71 (0.59, 0.85)	2.17	1.00 (reference)	0.60 (0.49, 0.75)	2.72
**Hemorrhagic stroke**						
No. of events (%)	275 (2.1%)	39 (1.6%)		50 (2.0%)	39 (1.5%)	
IR per 1000 person-years (95% CI)	4.70 (4.16, 5.29)	3.19 (2.27, 4.35)		4.73 (3.51, 6.24)	3.19 (2.27, 4.36)	
Unadjusted HR (95% CI)	1.00 (reference)	0.64 (0.46, 0.90)	2.50	1.00 (reference)	0.63 (0.42, 0.96)	2.55
Multivariable-adjusted HR (95% CI)	1.00 (reference)	0.66 (0.47, 0.92)	2.40	1.00 (reference)	0.63 (0.42, 0.96)	2.55
Propensity-score-adjusted HR (95% CI)	1.00 (reference)	0.63 (0.45, 0.88)	2.55	1.00 (reference)	0.64 (0.42, 0.97)	2.50

In the unadjusted model, the only treatment was included as a covariate. In the multivariable model, all additional covariates were included. Using the same covariates, the individual propensity score was calculated. IR, incidence rate; HR, hazard ratio; CI, confidence interval.

## Data Availability

All data supporting the findings are in the manuscript. More detailed information and raw data can be obtained from the corresponding author upon reasonable request.

## Supplementary Materials

The following supporting information can be downloaded at https://www.mdpi.com/article/10.3390/ph16010057/s1. Figure S1: absolute standardized difference of covariates before and after propensity score matching; Figure S2: association between the use of spironolactone and outcomes in other subgroups. (A) total stroke, (B) ischemic stroke, (C) hemorrhagic stroke; Figure S3: forest plot showing results of sensitivity analyses based on different analysis strategies; Figure S4: STROBE participant flow diagram; Table S1: sensitivity analyses by excluding subjects with ages of more than 80 years in propensity-score-matched cohort; Table S2: sensitivity analysis excluding outcome events within the first year of follow-up in propensity-score-matched cohort; Table S3: competing risk models for the association of spironolactone use with the risk of stroke in propensity-score-matched cohort; Table S4: incidence of hyperkalemia events in patients in the spironolactone-use and non-use groups after propensity score matching; and Table S5: list of medications included in the study.
