# Supplementary material for: Association between Use of Spironolactone and Risk of Stroke in Hypertensive Patients: A Cohort Study
Source: Pharmaceuticals (Basel). 2022 Dec 30;16(1):57. doi: 10.3390/ph16010057 (PMC9861555; doi:10.3390/ph16010057)
Supplement: Supplementary file 1 [file pharmaceuticals-16-00057-s001.zip › pharmaceuticals-2045747-supplementary.pdf]

Supplementary Material

1 Supplemental material and methods

Precise details of the PSM.

Propensity scores were the conditional probabilities of performing a specific exposure (spironolactone users versus non-users) given a set of baseline measurement covariates. Propensity scores were estimated using a non-parsimonious multivariate logistic regression model with spironolactone users as the dependent variable and all baseline characteristics listed in Table 1 as covariates. Matching was performed using a 1:1 matching protocol without replacement (greedy matching algorithm) with a caliper width equal to 0.2 of the standard deviation of the propensity score logit.

2 Supplementary Figures and Tables

2.1 Supplementary Figures

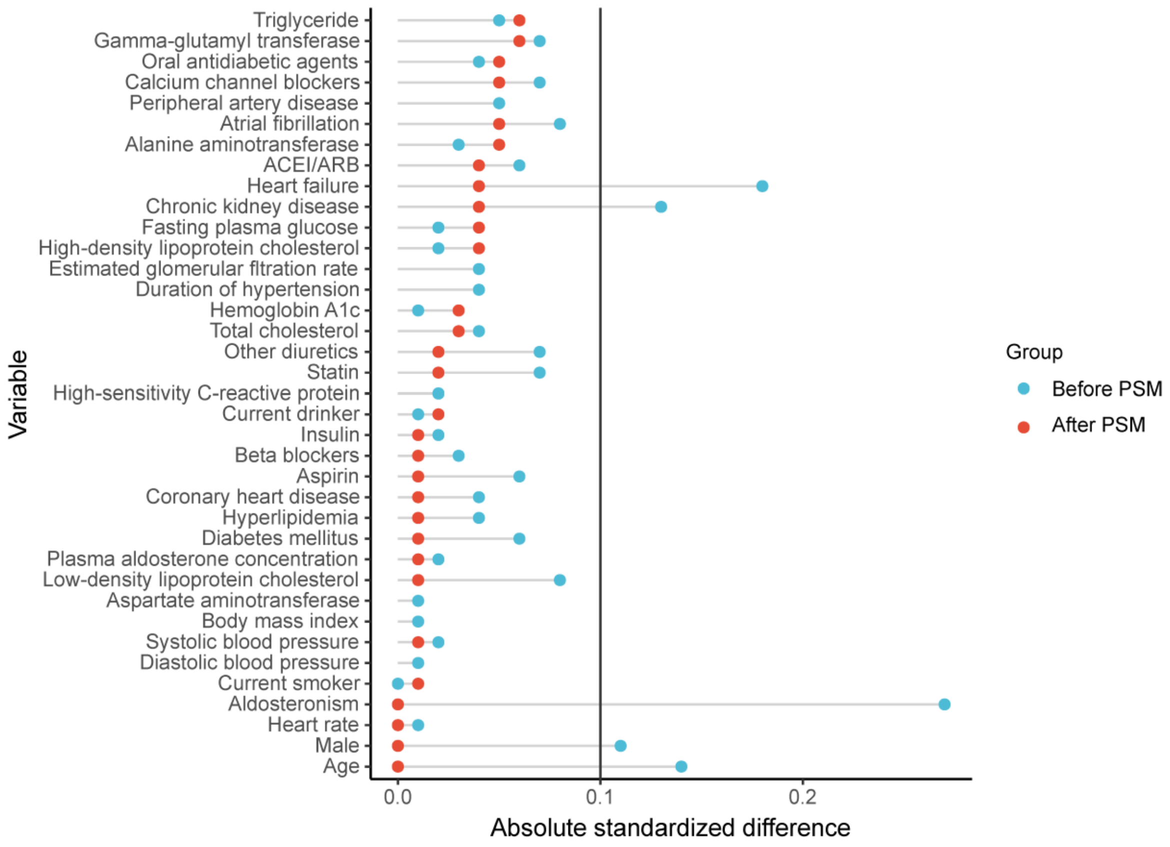

Figure S1. Absolute standardized difference of covariates before and after propensity score matching.

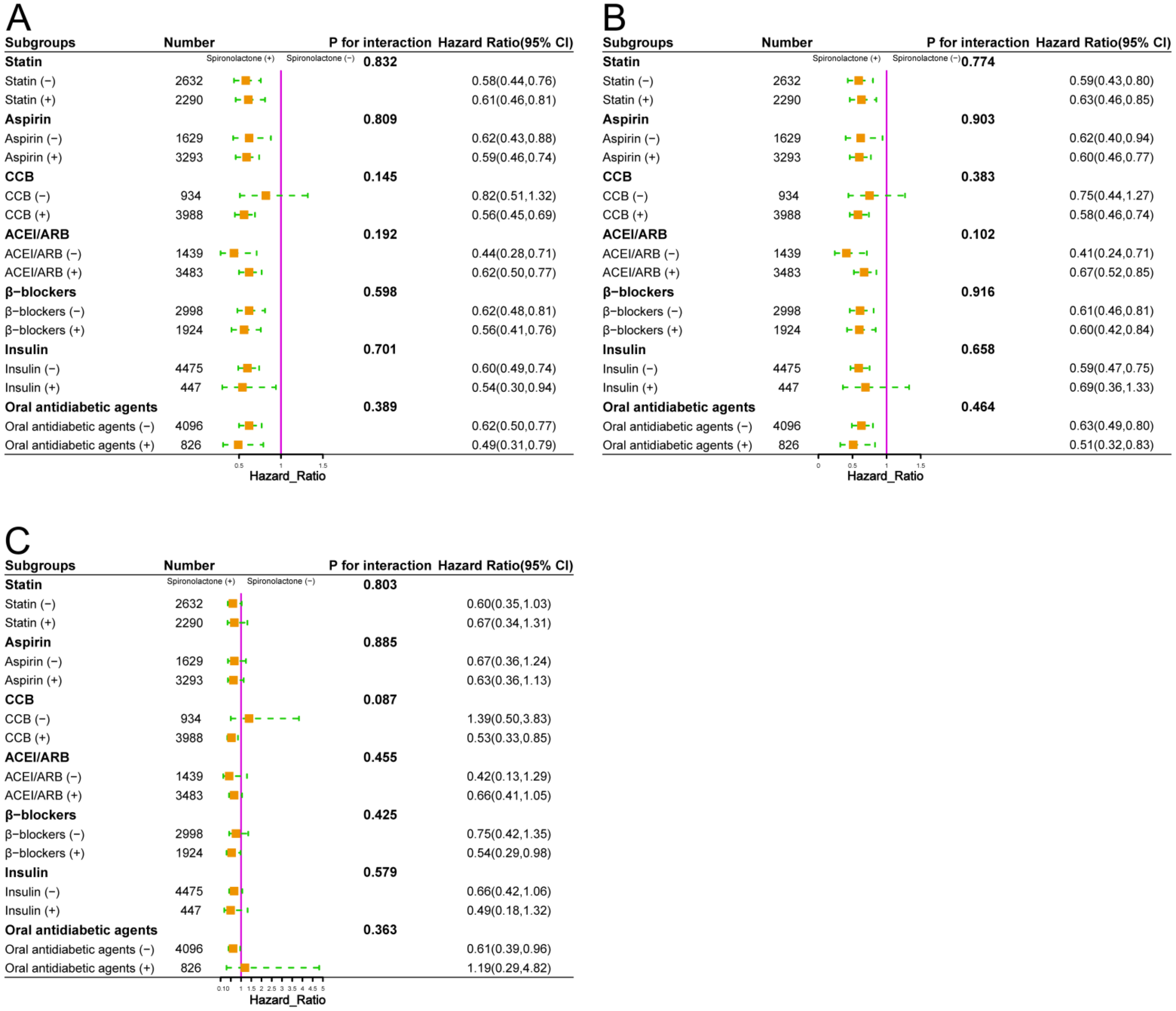

Figure S2. Association between the use of spironolactone and outcomes in other subgroups. (A) total stroke, (B) ischemic stroke, (C) hemorrhagic stroke.

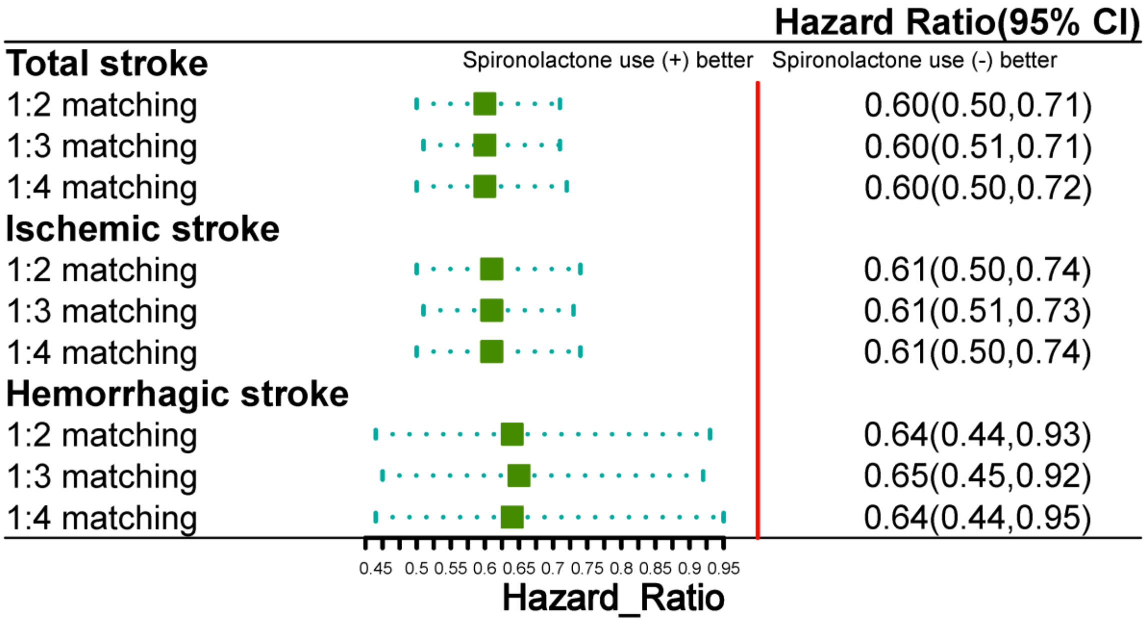

Figure S3. Forest plot showing results of sensitivity analyses based on different analysis strategy.

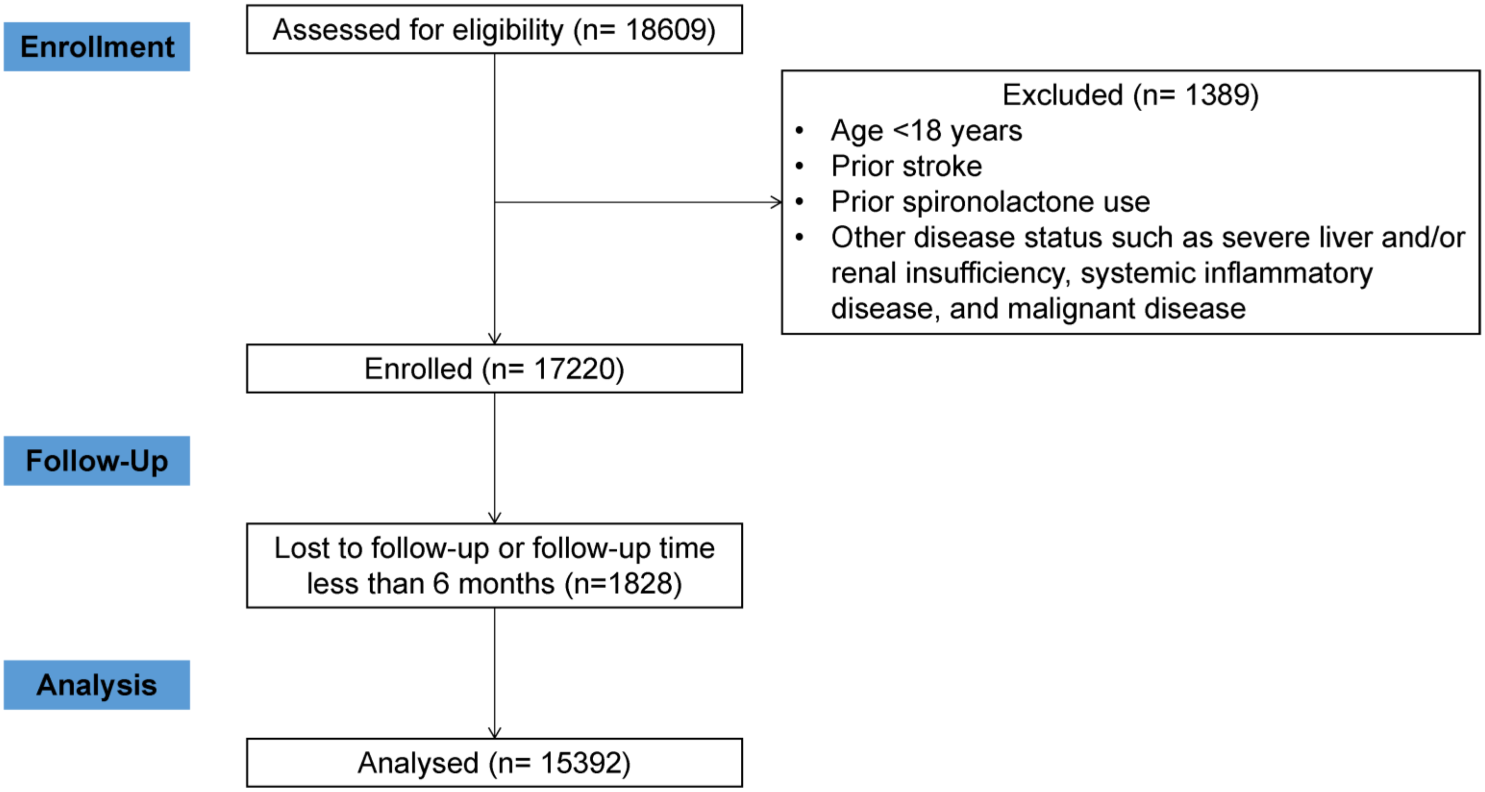

Figure S4. STROBE participant flow diagram.

2.2 Supplementary Tables

Table S1. Sensitivity analyses by excluding subjects with age more than 80 years in propensity score-matched cohort.

	Nonusers	Spironolactone users	P value
**Total stroke**			
Unadjusted HR (95% CI)	1.00 (reference)	0.58 (0.48, 0.71)	<0.001
Multivariable adjusted HR (95% CI)	1.00 (reference)	0.61 (0.50, 0.75)	<0.001
Propensity score-adjusted HR (95% CI)	1.00 (reference)	0.59 (0.48, 0.72)	<0.001
**Ischemic stroke**			
Unadjusted HR (95% CI)	1.00 (reference)	0.59 (0.47, 0.73)	<0.001
Multivariable adjusted HR (95% CI)	1.00 (reference)	0.62 (0.50, 0.78)	0.001
Propensity score-adjusted HR (95% CI)	1.00 (reference)	0.59 (0.47, 0.74)	<0.001
**Hemorrhagic stroke**			
Unadjusted HR (95% CI)	1.00 (reference)	0.64 (0.42, 0.97)	0.036
Multivariable adjusted HR (95% CI)	1.00 (reference)	0.64 (0.42, 0.97)	0.035
Propensity score-adjusted HR (95% CI)	1.00 (reference)	0.64 (0.42, 0.98)	0.040

Table S2. Sensitivity analysis excluding outcome events within the first year of follow-up in propensity score-matched cohort.

	Nonuser group	User group	P value
**Total stroke**			
Unadjusted HR (95% CI)	1.00 (reference)	0.64 (0.52, 0.79)	<0.001
Multivariable adjusted HR (95% CI)	1.00 (reference)	0.65 (0.53, 0.81)	<0.001
Propensity score-adjusted HR (95% CI)	1.00 (reference)	0.65 (0.52, 0.80)	<0.001
**Types of stroke**			
**Ischemic stroke**			
Unadjusted HR (95% CI)	1.00 (reference)	0.65 (0.52, 0.82)	<0.001
Multivariable adjusted HR (95% CI)	1.00 (reference)	0.67 (0.53, 0.84)	0.001
Propensity score-adjusted HR (95% CI)	1.00 (reference)	0.66 (0.52, 0.83)	<0.001
**Hemorrhagic stroke**			
Unadjusted HR (95% CI)	1.00 (reference)	0.70 (0.45, 1.09)	0.116
Multivariable adjusted HR (95% CI)	1.00 (reference)	0.71 (0.46, 1.10)	0.121
Propensity score-adjusted HR (95% CI)	1.00 (reference)	0.71 (0.46, 1.10)	0.124

In the unadjusted model only treatment was included as covariate. In the multivariable model 35 additional covariates were included. Using the same covariates, the individual propensity score were calculated.

HR, hazard ratio; CI, confidence interval.

Table S3. Competing risk models for the association of spironolactone use with the risk of stroke in propensity score-matched cohort.

	Nonusers	Spironolactone users	P value
**Total stroke**			
Subdistribution HR† (95% CI)	1.00 (reference)	0.60 (0.49, 0.74)	<0.001
**Types of stroke**			
**Ischemic stroke**			
Subdistribution HR† (95% CI)	1.00 (reference)	0.62 (0.49, 0.79)	<0.001
**Hemorrhagic stroke**			
Subdistribution HR† (95% CI)	1.00 (reference)	0.61 (0.39, 0.96)	0.034

†Adjusted Fine & Gray subdistribution hazard model accounting for competing risk of death attributable to other causes.

Table S4. Incidence of hyperkalemia events in patients in the spironolactone-use and non-use groups after propensity score matching.

Safety outcome	Unadjusted HR	Multivariable adjusted HR	Propensity score-adjusted HR
Nonuser vs User			
Nonuser group	1.00 (reference)	1.00 (reference)	1.00 (reference)
User group	1.21 (0.79, 1.87)	1.22 (0.79, 1.88)	1.23 (0.80, 1.90)
Average daily dose of spironolactone			
Nonuser group	1.00 (reference)	1.00 (reference)	1.00 (reference)
≤20mg/day	0.77 (0.41, 1.46)	0.77 (0.41, 1.46)	0.79 (0.42, 1.50)
40mg/day	1.28 (0.72, 2.26)	1.29 (0.73, 2.29)	1.27 (0.72, 2.25)
≥60mg/day	1.82 (1.03, 3.19)	1.83 (1.04, 3.22)	1.87 (1.07, 3.28)
Duration of spironolactone consumption			
Nonuser group	1.00 (reference)	1.00 (reference)	1.00 (reference)
≥0.5, <1 years	1.57 (0.69, 3.54)	1.56 (0.69, 3.53)	1.37 (0.60, 3.14)
≥1, <5 years	1.57 (0.91, 2.71)	1.60 (0.93, 2.76)	1.60 (0.93, 2.76)
≥5 years	0.94 (0.55, 1.60)	0.94 (0.55, 1.61)	0.98 (0.57, 1.68)

Table S5. List of medications included in the study.

Drug class	Drug name
Aspirin	Aspirin
Beta-blocker	Atenolol, bisoprolol, carvedilol, metoprolol, propranolol
Angiotensin-converting enzyme inhibitors or angiotensin receptor blockers	Azilsartan, candestartan, captopril, enalapril, fosinopril, irbesartan, losartan, olmesartan, ramipril telmisartan, valsartan
Calcium channel blockers	Amlodipine, diltiazem, felodipine, lercanidipine, nifedipine, verapamil
Spironolactone	Spironolactone
Other diuretics	Acetazolamide, amiloride, benzyl hydrochlorothiazide, bumetanide, furosemide, hydrochlorothiazide, indapamide
Statin	Atorvastatin, fluvastatin, pitavastatin, rosuvastatin, simvastatin
Oral antidiabetic agents	Metformin, glipizide, gliclazide, glimepiride, glyburide, alogliptin, linagliptin, sitagliptin, vidagliptin, saxagliptin, acarbose, nateglinide, meglitinide, repaglinde, pioglitzone,dulaglutide, exenatide, liraglutide
Insulin	Rapid, short, intermediate and long-acting insulins
